# Tetramer formation in *Arabidopsis* MADS domain proteins: analysis of a protein-protein interaction network

**DOI:** 10.1186/1752-0509-8-9

**Published:** 2014-01-27

**Authors:** Carlos Espinosa-Soto, Richard GH Immink, Gerco C Angenent, Elena R Alvarez-Buylla, Stefan de Folter

**Affiliations:** 1 Laboratorio Nacional de Genómica para la Biodiversidad (Langebio), Centro de Investigación y de Estudios Avanzados del Instituto Politécnico Nacional (CINVESTAV-IPN), Km 9.6 Libramiento Norte Carretera León, C.P. 36821 Irapuato, Mexico; 2 Current address: Instituto de Física, Universidad Autónoma de San Luis Potosí, Manuel Nava 6, Zona Universitaria, C.P. 78290 San Luis Potosí, Mexico; 3 Plant Research International, 6700 AA Wageningen, The Netherlands; 4 Laboratory of Molecular Biology, Wageningen University, 6700 AA Wageningen, The Netherlands; 5 Departamento de Ecología Funcional. Instituto de Ecología, Universidad Nacional Autónoma de México, Ap. Postal 70-275, 3er Circ. Ext. Jto. Jard. Bot., CU, C.P. 04510 Mexico, D.F., Mexico

**Keywords:** MADS domain proteins, Protein-protein interaction network, Transcription factors, Tetramers, Subgraph abundance, *Arabidopsis thaliana*

## Abstract

**Background:**

MADS domain proteins are transcription factors that coordinate several important developmental processes in plants. These proteins interact with other MADS domain proteins to form dimers, and it has been proposed that they are able to associate as tetrameric complexes that regulate transcription of target genes. Whether the formation of functional tetramers is a widespread property of plant MADS domain proteins, or it is specific to few of these transcriptional regulators remains unclear.

**Results:**

We analyzed the structure of the network of physical interactions among MADS domain proteins in *Arabidopsis thaliana*. We determined the abundance of subgraphs that represent the connection pattern expected for a MADS domain protein heterotetramer. These subgraphs were significantly more abundant in the MADS domain protein interaction network than in randomized analogous networks. Importantly, these subgraphs are not significantly frequent in a protein interaction network of TCP plant transcription factors, when compared to expectation by chance. In addition, we found that MADS domain proteins in tetramer-like subgraphs are more likely to be expressed jointly than proteins in other subgraphs. This effect is mainly due to proteins in the monophyletic MIKC clade, as there is no association between tetramer-like subgraphs and co-expression for proteins outside this clade.

**Conclusions:**

Our results support that the tendency to form functional tetramers is widespread in the MADS domain protein-protein interaction network. Our observations also suggest that this trend is prevalent, or perhaps exclusive, for proteins in the MIKC clade. Because it is possible to retrodict several experimental results from our analyses, our work can be an important aid to make new predictions and facilitates experimental research on plant MADS domain proteins.

## Background

MADS domain proteins comprise a family of eukaryotic transcription factors that are very important for plant and animal development [1-4]. In plants, MADS domain transcription factors have diversified extensively after multiple independent duplication events, and they now play important roles in many aspects of development [2,5,6]. They are involved in many processes, such as floral timing, floral organ identity specification, gametophyte, fruit and root development, among others [2,7-10]. The importance of plant MADS domain proteins is reflected in their evolutionary history: some events in the history of this protein family correlate with major evolutionary modifications in plant morphogenesis. Moreover, there is evidence of positive selection having acted at particular aminoacids of these plant proteins at the times of these evolutionary events [5,6,11].

MADS domain proteins are grouped into two distinct monophyletic clades, type I and type II, that split before the separation of animal and plant lineages [12]. Each of these two clades includes smaller sub-clades [4]. Within type II proteins, the plant-specific MIKC monophyletic clade stands out. MIKC proteins have a distinctive structure in which four different domains have been recognized. The first is the MADS (M) domain, that is widely conserved, common to all MADS domain proteins, and participates in binding to DNA. MIKC proteins also have a poorly conserved Intervening (I) domain, which is important for the specificity of dimer formation. Next we find a Keratin-like (K) domain that is involved in protein-protein interactions. Finally, there is a C-terminal (C) domain that may carry transcriptional activation domains. Most MADS domain proteins that are functionally characterized belong to the MIKC clade [6,7,9].

Importantly, MADS domain proteins do not exert their functions as monomers, but rather they form multimeric protein complexes with other MADS domain proteins [13-16]. A comprehensive understanding of how MADS domain proteins interact with multiple molecules in a concerted manner would provide a significant advance in understanding the molecular basis of the wide range of developmental mechanisms in which these proteins participate.

At least some MADS domain proteins build tetramers with other MADS domain proteins to regulate transcription of their target genes, as proposed originally by the ‘quartet model’ [17,18]. The tetramer binds two different sites in the regulatory sequence of target genes and forces looping of DNA stretches, consequently regulating the transcription of the associated genes [19-21]. For instance, the floral homeotic MADS domain proteins APETALA3 (AP3) and PISTILLATA (PI) bind two other MADS domain proteins, APETALA1 (AP1) and SEPALLATA3 (SEP3), to determine petal cell identity in the flower of the thale cress *Arabidopsis thaliana*[22-24]. It is noteworthy that, so far, all experimentally validated tetramers comprise proteins from the MIKC clade exclusively. It is still unknown if tetramer formation is a widespread property of many plant MADS domain proteins, or if it is specific to a few of them.

Here we address if tetramer formation is widespread among MADS domain proteins in the plant model system *Arabidopsis thaliana*. We do so by analyzing the structure of a network of physical interactions among *Arabidopsis* MADS domain proteins. We built the network with data from high-throughput yeast two and three-hybrid studies that have uncovered a near-complete map of interactions among MADS domain proteins in *Arabidopsis*[15,25]. We supplemented these data with additional information from various sources and analyzed the resulting protein-protein interaction network.

First, we studied the abundance of different subgraphs in the network. Each subgraph represents a pattern of connections among proteins in a set, in a manner independent of the identity of each protein. The abundance of particular subgraphs in different kinds of biological networks may indicate that natural selection has favored such a connection pattern across evolution. This would be the case, for example, when elements connected in that manner jointly perform a function that confers an advantage to an organism. Consider the case of biological networks where transmission of information is important, such as neuron nets or signal transduction networks. In this kind of networks a particular subgraph, known as feedforward loop, appears with a frequency higher than expected by chance [26-28]. Such a connection pattern is seemingly useful to perform distinct signal-processing tasks [29,30].

If the ability to build functional tetramers is a widespread property of plant MADS domain transcription factors, we expect that subgraphs compatible with tetramer formation will appear more often than expected by chance in the MADS-domain protein-protein interaction network. Experimental research has provided valuable information regarding how some MADS domain proteins bind other such proteins to form tetramers. These studies thus hint on the properties of the subgraphs that could represent this kind of protein complexes. Apparently, each MADS domain protein binds two other proteins in the tetramer through different protein domains. A MADS domain protein binds, through one of its domains, a partner to form a dimer. It also establishes another interaction, through a different domain, to another MADS domain protein in a second dimer. For example, consider the experimental analyses of the roles of different parts of AGAMOUS (AG), another floral homeotic MADS domain protein. Such studies have shown that two adjacent regions of AG, the MADS domain and I region, are sufficient for DNA binding and for the formation of either homodimers [31] or heterodimers with the MADS domain protein SEPALLATA1 (SEP1) [32]. However, these two protein regions are not sufficient for a functional AG protein. The C region is also required, most likely for binding other proteins to form higher-order protein complexes [31]. Indeed, Fan and collaborators found that the K and C domains of AG can bind other MADS domain proteins, such as AGAMOUSLIKE-6 (AGL6), SEP1, SEPALLATA2 or SEPALLATA3 (SEP1-3) [33]. Another example concerns the three predicted *α*-helices (K1, K2, and K3) in the K domain of the PI protein. While K1 is more important for the PI-AP3 interaction, K3 is more important for PI’s interaction with SEP3 [34]. Finally, Melzer et al. have shown that a part of the K domain in the MADS domain protein SEPALLATA3 (SEP3) is not essential for the formation of SEP3 homodimers [21]. However, the same SEP3 K domain is required to mediate interactions between a SEP3 homodimer and the APETALA3-PISTILLATA heterodimer [20], or another SEP3 homodimer [21].

The experimental evidence mentioned in the preceding paragraph suggests that functional MADS domain tetramers require that each protein binds, through different protein regions, two other proteins in the tetramer. Thus, we paid special attention in our analysis to those four-node subgraphs where each node interacts with two other nodes in the subgraph. We found that the network of protein-protein interactions among MADS domain proteins in *Arabidopsis thaliana* contains significantly more such tetramer-compatible subgraphs than expected by chance. In contrast, tetramer-compatible subgraphs are not significantly abundant in an analogous network for a different family of plant transcription factors, the TCP protein family [35,36]. Moreover, MADS domain proteins in a tetramer-compatible subgraph are more likely to be co-expressed than proteins in other subgraphs. In addition, we show that this association between tetramer-like subgraphs and joint expression is evident only for proteins in the MIKC clade. We also analyzed several aspects of the organization of the whole network of interactions among MADS domain proteins. Taken together, our results suggest that the formation of tetramers is a widespread property of *Arabidopsis* MIKC MADS domain proteins. We finally discuss how our work may be an important aid towards the design of new experiments to uncover functional tetramers *in planta*.

## Results

### A network of interactions among MADS domain proteins

**Figure 1:**
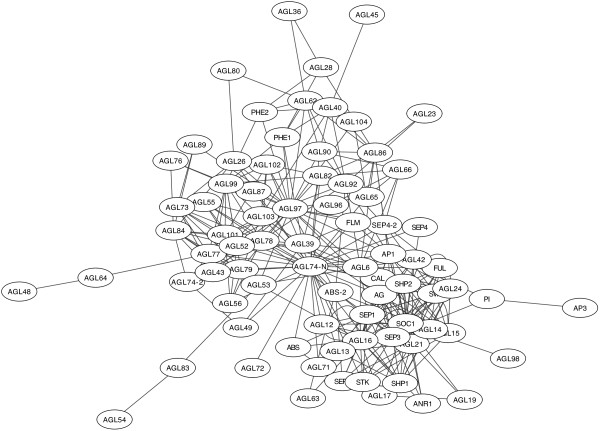
**Network of protein-protein interactions among *Arabidopsis *MADS domain proteins.** Each node represents an *Arabidopsis* MADS domain protein encoded by a different gene. Edges bind pairs of nodes that physically interact.

In order to study the organization of interactions between MADS domain proteins in the plant *Arabidopsis thaliana*, we assembled a network of interactions of these proteins. In such a network, nodes represent distinct MADS domain proteins and edges represent the potential of two proteins to physically interact, according to published experimental information. We started by including protein-protein interactions found by a set of yeast two-hybrid assays [15]. We also considered interactions obtained through a large-scale study based on yeast three-hybrid analyses [25], as described in Methods. The network was supplemented with information from several other sources that have established interactions between *Arabidopsis* MADS domain proteins by means of analyzing small sets of proteins [21-23,33,37] (see Table S1 in Additional file 1). Figure 1 shows the resulting non-directed network. From now on we refer to this network as the MADS network.

The MADS network comprises 79 nodes, including four proteins that result from alternative splicing (ABS-2, SEP4-2, AGL74-2 and AGL74-N). There are 312 protein-protein interactions in the network. The most highly connected node (AGL74-N) has 34 interactions, and the median number of interactions per protein is 6.

### Some connection patterns appear with a frequency higher than expected by chance in the MADS network

**Figure 2:**
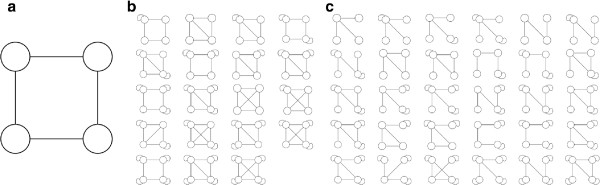
**Subgraphs composed of four nodes. a**. Subgraph that represents a connection pattern associated to MADS domain protein heterotetramers. Each node binds two other nodes in this subgraph. **b**. Subgraphs that include all those interactions in **a**. Curved edges with both ends on the same node represent interactions between identical copies of a protein. **c**. Subgraphs that do not include all the interactions in **a**.

Despite a great experimental effort, we still do not know how widespread tetramer formation is in the *Arabidopsis* MADS domain transcription factor family. As we state in the introduction, diverse sources of evidence suggest that tetramer formation among MADS domain proteins depends on each MADS domain protein binding directly to two other MADS domain proteins in the tetramer through different protein domains. The subgraph schematized in Figure 2a represents such a connection pattern.

For each of the 50 possible ways of connecting *N* = 4 nodes (Figure 2), we determined how many instances of that subgraph occurred in the MADS network. In order to find out whether the abundance of the different connection patterns in the MADS network is significant, we compared our counts to those of randomized networks that we used as reference. Each of the 10^4^ randomized networks that we built had the same number of nodes, connections and connectivity distribution as the MADS network (see Methods). After correcting for multiple hypotheses testing [38], we found five connection patterns that were significantly more abundant in the MADS network than in the randomized networks (Table 1). In fact, for each of these five connection patterns there was not a single of the 10^4^ sampled random networks with at least the same number of instances of the connection pattern as the MADS network. The subgraph that we expect to be related to tetramers appears among these five subgraphs (second row in Table 1). Moreover, it is noteworthy that, of all the significantly abundant connection patterns in Table 1, only one (first row) does not include all the interactions present in the subgraph in Figure 2a. In sum, four out of five of the overrepresented connection patterns are compatible with the formation of heterotetramers. Taken together, our results suggest that there is a trend to form tetramers in the MADS-domain protein-protein interaction network. We further pursued this hypothesis in several ways.

**Table 1:** Abundance of frequent subgraphs in the MADS network as compared to that of randomized networks

**Subgraph**	***N***_***MADS***_	**Randomized networks**				***p*-value**
		**Mean**	**Median**	**Std. dev.**	**Std. error**	
	9929	2324.41	2035	1319.16	13.1916	<10^-4^
	891	118.989	103	71.7229	0.717229	<10^-4^
	336	140.91	142	51.2349	0.512349	<10^-4^
	133	31.2057	30	11.3476	0.113476	<10^-4^
	93	17.0886	16	11.0623	0.110623	<10^-4^

First, we considered all those connection patterns that contain the interactions in the subgraph that is significantly abundant in the MADS network but that is not compatible with tetramer formation (first row in Table 1). In other words, we counted those subgraphs in which nodes have at least the links in that connection pattern (first row in Table 1), but possibly also some other additional links. We found that subgraphs that embed such a pattern are not more abundant than as expected by chance (first row in Table 2). In contrast, subgraphs that include, at least, all the interactions in the tetramer-compatible pattern (Figure 2a,b) are more frequent in the MADS network than in randomized networks (fifth row in Table 2). Moreover, it is noteworthy that connection patterns that embed subgraphs distinct to the subgraph in Figure 2a, but similar in arrangement or number of interactions, are not more abundant in the MADS network than as expected by chance (rows 2–4 in Table 2). From now on, we will refer to connection patterns that incorporate (perhaps not exclusively) the interactions in the subgraph in Figure 2a as tetramer-like subgraphs or connection patterns. Such connection patterns are listed in Figure 2a,b. Remarkably, none of 10^4^ sampled randomized networks had at least as many tetramer-like subgraphs as the MADS network (last row in Table 2).

**Table 2:** Abundance of connection patterns in the MADS network

**Embedded subgraph**	***N***_***MADS***_	**Randomized networks**				***p*-value**
		**Mean**	**Median**	**Std. dev.**	**Std. error**	
	19171	18729.6	18670	602.99	6.0299	0.2188
	19965	24132.9	24133	592.969	5.92969	1
	6499	7412.84	7409	433.905	4.33905	0.9837
	370	487.759	421.5	348.13	3.4813	0.5619
	2751	1804.51	1802	113.224	1.13224	<10^-4^

Table shows results for connection patterns that forcefully include some interactions (solid lines) but may or not contain other interactions (dashed lines).

Throughout this work we have assumed that most positive results in yeast-three hybrid assays reflect pairwise protein-protein interactions. Consider an assay where proteins *X* and *Z* function as bait and prey, respectively, and that produces a positive result only in the presence of a third protein *Y*. In this case, we assume pairwise interactions between *X* and *Y* on the one hand, and between *Y* and *Z*, on the other (see Methods). Alternatively, such a positive result could indicate that these proteins bind only in the presence of all three proteins. We have dismissed the latter alternative, as a simplifying assumption. Nonetheless, it is noteworthy that more than two thirds (58/84) of the pairwise interactions that we derived from a large scale three-hybrid study [25] were also supported by two-hybrid data in the study by de Folter and collaborators [15]. Moreover, if the formation of higher-order complexes, as those inferred from yeast three-hybrid studies, depended on non-pairwise interactions between a dimer and a third protein there would be specific implications that are not fulfilled. Namely, if the aforementioned hypothetical proteins *X* and *Y* form the dimer that binds in a non-pairwise manner to protein *Z*, we would also recover the interaction between *X* and *Y* from yeast two-hybrid studies, but not the interaction between *Y* and *Z*. In other words, a scenario where non-pairwise interactions are prevalent in higher-order protein complexes implies that, for most positive yeast three-hybrid results, only one pairwise interaction is also supported by yeast two-hybrid data. In contrast, our assumption predicts that for most positive yeast three-hybrid results, there are two interactions supported by yeast two-hybrid assays. We thus assessed how many of the sets of three proteins that produce a positive yeast three-hybrid in a large-scale study [25] reflect these patterns in the large-scale two-hybrid study [15]. Of the 116 sets of three proteins that yield a three-hybrid positive result, 86 (74.1%) have two interactions also supported by two-hybrid data, consistently with our hypothesis. In contrast, 26 (22.4%) and four (3.4%) have only one or none interactions, respectively, supported by two-hybrid data.

Our observations suggest that most interactions inferred from yeast three-hybrid data are pairwise, as we assumed. Notwithstanding, we addressed whether our observations in Tables 1 and 2 depend on this assumption. We assembled a new network that includes only interactions from the aforementioned two-hybrid study [15]. This network thus contains only interactions confirmed to be pairwise. We compared subgraph counts of this network to that of 10^4^ of randomized networks that preserved the number of nodes, connections, and connectivity distribution. The results are qualitatively the same to those that we obtained from the analysis of the MADS network (compare Tables S2 and S3 in Additional file 1 to Tables 1 and 2, respectively). Thus, the statistically significant abundance of tetramer-like connection patterns in the MADS network does not depend on the assumption that interactions derived from three-hybrid data are pairwise. In conclusion, our results on the analysis of the MADS network are robust to the removal of all the interactions derived from three-hybrid data.

### Tetramer-like subgraphs are not abundant in interaction networks of the TCP plant protein family

It may be that the abundance of tetramer-like connection patterns is not specific of MADS domain proteins, but rather a general trend in plant protein interaction networks. If this is the case, the abundance of tetramer-like subgraphs would be more easily explained by factors common to the evolution of plant protein interaction networks rather than by functional constraints favoring MADS domain proteins to form tetrameric complexes. Secondly, the observed abundance of tetramer-like connection patterns could be due to the nature of the yeast-based protein-protein interaction assay. In this case, similar trends and distribution of subgraphs are expected in a large-scale yeast n-hybrid data set for another protein family.

**Figure 3:**
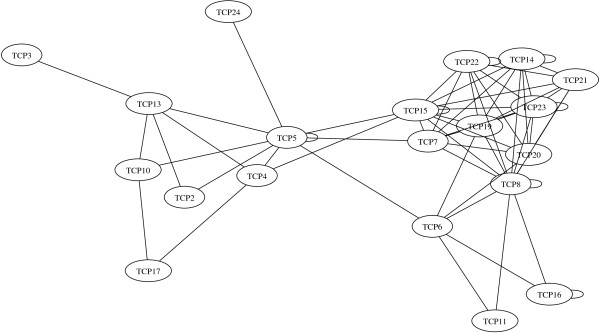
**Network of protein-protein interactions among *Arabidopsis *TCP proteins.** Each node represents a TCP protein encoded by a different gene. Edges bind pairs of nodes that physically interact.

To address the above two possibilities, a protein interaction network for another family of plant transcription factors was analyzed. We considered TCP proteins in *Arabidopsis*. These are transcription factors important for the regulation of different growth processes [35]. We extracted interactions between TCP proteins from a large-scale yeast two-hybrid study [36,39]. The TCP network contains 62 interactions among 20 nodes (Figure 3). We built 10^4^ randomized networks with the same number of nodes, connections and the same degree distribution as the original TCP network, analogously to what we did for the MADS network. We then counted how many times tetramer-like connection patterns featured in the TCP and randomized networks. The TCP network did not show significantly abundant tetramer-like subgraphs, when compared to the randomized networks (Table 3). Our observations on the TCP network are consistent with the hypothesis that the abundance of tetramer-like subgraphs that we observed in the MADS network is not a property of all plant transcription factor interaction networks. Thus, the abundance of such subgraphs may well be a functional constraint associated to MADS domain protein complexes.

**Table 3:** Abundance of tetramer-like connection patterns in the TCP network as compared to that of randomized networks

**Subgraph**	***N***_***TCP***_	**Randomized networks**				***p*-value**
		**Mean**	**Median**	**Std. dev.**	**Std. error**	
	169	161.103	160	22.977	0.22977	0.362

The numbers of proteins and interactions in the TCP and MADS networks are so contrasting that one may think that network size could underlie the differences in subgraph abundance that we found between these two networks. This is unlikely, since we compare each network with randomized versions with the same number of nodes, connections, and connectivity distribution. Notwithstanding, we addressed this possibility by sampling randomly 20-node sub-networks from the MADS network, keeping only those sub-networks that had the same connectivity distribution as the TCP network. In that manner, from a sample of more than 10^6^ sets of MADS domain proteins, we obtained 50 sub-networks. These sub-networks contained experimentally validated interactions among MADS domain proteins, but also the same number of nodes and connectivity distribution as the TCP network. The 50 sub-networks taken from the MADS network have an average of 216.22 tetramer-like connection patterns (median = 216.5; std. dev. = 19.37). In contrast, the 10^4^ randomized networks have a mean number of only 161.1 tetramer-like connection patterns (median = 160; std. dev. = 22.9). The difference is statistically significant, according to a Mann-Whitney U test (*U* = 11.39; *p* < 2.6 × 10^-308^). In sum, tetramer-like connection patterns are significantly abundant in partitions of the MADS network with the same connectivity distribution as the TCP network, but not in the TCP network.

### Co-expression is frequent for MADS domain proteins in tetramer-like connection patterns

In order to form functional tetrameric complexes, MADS domain proteins in a tetramer-like connection patterns must be expressed jointly *in vivo*. To address whether this is usually the case, and also whether there is a difference in the degree of co-expression with proteins in other four-node subgraphs, we considered gene expression data reported in the developmental data set of the AtGenExpress project [40] (see Methods).

Several genes encoding for proteins in the MADS network lack expression data in the AtGenExpress data set. We thus built a new network by discarding such proteins from the MADS network. We call the resulting network the MADS_*exp*_ network. The MADS_*exp*_ network contains 54 nodes and 167 interactions. While the original MADS network contains 2751 different sets of proteins in tetramer-like subgraphs, the MADS_*exp*_ network includes 1351 such sets. Nevertheless, the MADS_*exp*_ network contains significantly more tetramer-like subgraphs than randomized networks with the same number of nodes, number of interactions, and degree distribution (first row in Table 4). In fact, this result holds after discarding those subgraphs composed of nodes (proteins) whose encoding mRNA is never co-expressed in at least one tissue (second row in Table 4).

**Table 4:** **Abundance of tetramer-like subgraphs in the MADS**_***exp ***_**network as compared to that of randomized networks**

	$N_{M_{\text{exp}}}$	**Randomized networks**				***p*-value**
		**Mean**	**Median**	**Std. dev.**	**Std. error**	
All tetramer-like subgraphs	1351	757.379	755	59.0441	0.590441	<10^-4^
Co-expressed tetramer-like subgraphs	1134	563.745	561	53.9929	0.539929	<10^-4^

We repeated the analyses described in the previous paragraph, but using the At-TAX data base [41], instead of the AtGenExpress data set. The At-TAX database resulted from a whole genome tiling array for *Arabidopsis*. It contains expression data for more genes, but for fewer tissues, than the AtGenExpress data set. We call the network that resulted after elimination of proteins lacking expression data in this set the MADS_*At*-*TAX*_ network. It contains 74 nodes and 254 edges. This network also has a significant abundance of tetramer-like connection patterns, when compared to randomized networks with the same connectivity distribution. Again, this result holds after dismissing those subgraphs including proteins whose encoding mRNA never coincides in at least one tissue. Despite the At-TAX database contains expression data for more genes encoding MADS domain proteins, it is less suitable than the AtGenExpress data set for the analysis of co-expression of different proteins. Given that the At-TAX database includes information for only ten different wild-type plant tissues, there are many pairs of co-expression false negatives. Consider, for instance, those sets of four proteins connected according to a tetramer-like connection pattern. The MADS_*exp*_ network contains 1351 such sets and the MADS_*At*-*TAX*_ contains 1849 of them. However, after dismissing those sets where evidence of co-expression is lacking, the MADS_*exp*_ network contains 1134 but the MADS_*At*-*TAX*_ includes only 574 of them. Thus, we performed the remaining analyses only using the AtGenExpress data set and the MADS_*exp*_ network.

**Figure 4:**
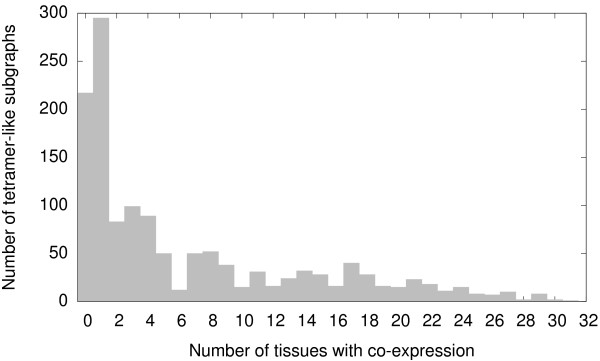
**Joint expression of genes encoding for proteins in tetramer-like subgraphs.** The genes that encode for the four proteins in most tetramer-like subgraphs are expressed jointly in one or more *Arabidopsis* tissues.

As mentioned above, we found in our analysis of the MADS_*exp*_ network that the mRNA encoding for proteins in most tetramer-like subgraphs is co-expressed in at least one *Arabidopsis* tissue: Of 1351 4-protein sets in tetramer-like subgraphs, the four genes encoding for the proteins of only 217 such sets are never co-expressed at the same time, based on the AtGenExpress data. However, for each of the remaining 1134 sets, the genes encoding the four constituent proteins are co-expressed in one or more tissues (Table 4 and Figure 4). The list with these 1134 protein sets is presented in Additional file 2, and we refer to each of such sets as a *prospective tetramer*. Thus, for proteins in tetramer-like subgraphs, the ratio of jointly expressed encoding genes (in at least one tissue) versus never co-expressed encoding genes is 5.23:1 (1134:217). The same ratio but for four-node subgraphs that are not tetramer-like is less than half as low (2.31:1 ; 4388:1896). The difference is statistically significant: A Pearson’s *χ*^2^ test rejects the null hypothesis that there is no association between joint expression of proteins (in at least one tissue) and formation of tetramer-like connection patterns (*χ*^2^ = 110.6; *df* = 1; *p* = 7.25 × 10^-26^). Thus, genes encoding for proteins in tetramer-like subgraphs are more likely to be co-expressed than genes encoding for proteins in other subgraphs.

**Figure 5:**
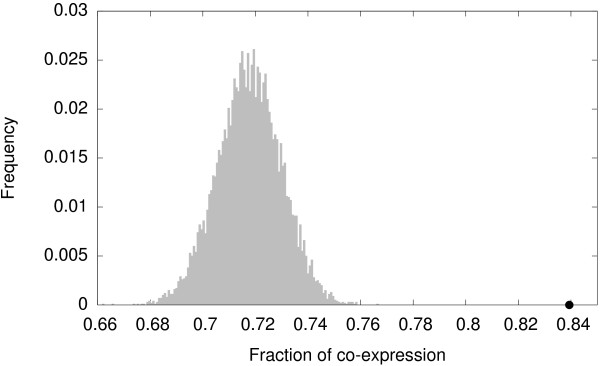
**The fraction of co-expression in the MADS**_***exp ***_**network is higher for tetramer-like subgraphs than for random ensembles of subgraphs.** We define the fraction of co-expression as the proportion of subgraphs composed of proteins whose genes are expressed jointly in at least one tissue. The black dot indicates the fraction of co-expression for tetramer-like subgraphs (0.84). The histogram represents the frequency distribution of the fraction of co-expression for a sample of 10^4^ ensembles, each composed of 1351 4-node subgraphs picked randomly from the MADS_*exp*_ network.

As a means of illustrating how large the differences in joint expression are between those genes encoding for proteins in tetramer-like subgraphs and those encoding for proteins in other subgraphs, we collected the data shown in Figure 5. We first assessed, for the 1351 protein sets in tetramer-like connection patterns, the fraction of sets where the encoding genes are co-expressed in at least one tissue. This fraction equals 0.84 (1134/1351). We then picked 10^4^ samples, each composed of 1351 subgraphs taken randomly from the 7635 4-node subgraphs in the MADS_*exp*_ network. For each such sample, we determined the fraction of sets where all the four protein-encoding genes are co-expressed in one or more tissues. Joint expression is more frequent in tetramer-like subgraphs than in random subgraphs in the MADS_*exp*_ network. In fact, none of the 10^4^ samples had a fraction of co-expression as high as that of tetramer-like subgraphs (*p*<10^-4^).

**Figure 6:**
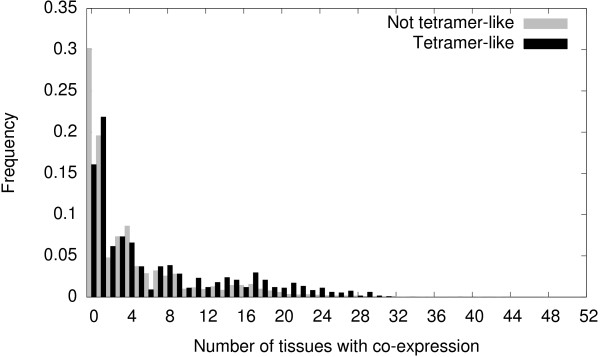
**Distributions of protein sets co-expressed in *n *tissues.** The distribution for protein sets in tetramer-like subgraphs has a broader tail than that of the distribution associated to subgraphs that are not tetramer-like.

Additional evidence suggests that, in terms of MADS-box gene expression, tetramer-like connection patterns are distinct from other subgraphs. We considered the distribution of all the connected 4-node subgraphs in the MADS_*exp*_ network expressed in *n* tissues, distinguishing between those sets of proteins in tetramer-like subgraphs and those in non-tetramer-like subgraphs. Figure 6 evidences that the distribution of tetramer-like subgraphs has a broader right tail. Indeed, a Mann-Whitney U test reveals that there are significant differences in the number of tissues where proteins in tetramer-like subgraphs or in other subgraphs are jointly expressed (*U* = 148.56, *p* < 2.6 × 10^-308^). In sum, it is easier to find a set of four MADS domain proteins whose genes are co-expressed in many tissues if the proteins are linked in a tetramer-like connection pattern than when linked in any other manner.

Recent experimental research suggests that SEP1-3 proteins have an important role facilitating the formation of MADS domain protein tetramers [20,21,25]. The composition of the list of prospective tetramers supports the prominence of these proteins for the assembly of tetrameric complexes: SEP1-3 proteins appear 840 times in the list of prospective tetramers. While SEP3 and SEP1 appear each 385 times, SEP2 appears only 70 times. It is noteworthy that other MADS domain proteins also occur many times in the list of prospective tetramers. AGL21 appears 376 times, SOC1 457 times, AGL6 290 times and AGL24 278 times. SEP1-3 proteins perform their functions in the floral meristem [42]. It may well be that other proteins that appear recurrently in the list of prospective tetramers play a SEP-like role in the formation of tetramers outside the flower.

### Proteins in prospective tetramers are mostly MIKC MADS domain proteins

Until now, all experimentally validated MADS domain protein tetramers consist exclusively of proteins in the MIKC clade [20-24]. The K domain appears exclusively in MIKC proteins [4,12] and, importantly, it has been implicated in several protein-protein interactions [14,20,21,33,34,43]. For these reasons, it may well be that the formation of MADS domain protein tetramers occurs mainly for MIKC proteins. We thus addressed whether this could be the case.

We found that, of all the 2751 tetramer-like connection patterns that the MADS network contains, 1397 contain only MIKC proteins and 1354 contain at least one non-MIKC protein. Of the latter, 773 comprise a mixture of MIKC and non-MIKC proteins, and the remaining 581 contain non-MIKC proteins exclusively. Remarkably, the story is very different when we analyze the protein sets in the list of prospective tetramers, which includes the 1134 protein sets in tetramer-like subgraphs that are expressed jointly in at least one tissue. In this case, there are 1127 protein sets that consist purely of MIKC proteins, but only seven sets with at least one non-MIKC protein (Table 5). Indeed, among proteins in tetramer-like subgraphs, there is a clear association between MIKC-only sets and joint expression, according to a Pearson’s *χ*^2^ test (*χ*^2^ = 1823.3; *df* = 1; *p* < 3.7×10^-235^).

**Table 5:** Tetramer-like subgraphs composed of MIKC proteins exclusively are more likely to be jointly expressed

**Protein set**	**Not co-expressed tetramer-like subgraphs (*T*)**	**Co-expressed tetramer-like subgraphs (*C*)**	***C*/*T***
Including non-MIKC	1347	7	0.005
Only MIKC	270	1127	4.174

Although our results support the formation of functional tetramers for MIKC proteins but not for non-MIKC proteins, two confounding factors must be first taken into account. The first is that the AtGenExpress data set includes expression data for a larger fraction of MIKC proteins when compared to non-MIKC proteins. The MADS network comprises 35 MIKC proteins and 44 non-MIKC proteins. In contrast, the MADS_*exp*_ network, in which we discarded proteins encoded by genes lacking expression data, includes 32 MIKC but only 23 non-MIKC proteins. We thus performed an additional analysis to control for this factor. In this analysis, we first sampled randomly 15 non-MIKC and 15 MIKC proteins from the MADS_*exp*_ network. The AtGenExpress data set contained expression information for all 30 genes encoding for such proteins. We next counted the number of sets of four of these 30 proteins where: i) the four proteins interacted according to a tetramer-like connection pattern, and ii) the genes encoding for such four proteins were co-expressed in at least one tissue. We performed 10^4^ independent samplings. Even though the number of MIKC and non-MIKC proteins was the same in each sample, we found immense differences in the number of MIKC and non-MIKC prospective tetramers. On average, in each sample we found 0.6 prospective tetramers composed exclusively of non-MIKC proteins (median = 0, std. dev. = 0.94). In contrast, we found a mean number of 49.1 prospective tetramers comprising only MIKC proteins (median = 37, std. dev. = 44.14). We also counted prospective tetramers comprising both MIKC and non-MIKC. In this case we found only an average of 0.36 (median = 0, std. dev. = 0.64). The difference between the abundance of MIKC-only and non-MIKC-only prospective tetramers is highly significant, according to a Mann-Whitney U test (*U* = 117.5; *p* < 2.6 × 10^-308^).

A second factor that may be confounding is that MIKC proteins tend to have a significantly broader mRNA expression pattern than non-MIKC proteins. According to AtGenExpress data, the mRNA of MIKC proteins in the MADS_*exp*_ network is found in a mean number of 29.5 tissues (median = 27, std. dev. = 18.6). In comparison, there is expression of the mRNA of non-MIKC proteins in an average of 10.9 tissues (median = 5, std. dev. = 17.1). The difference is statistically significant (Mann-Whitney *U* = 4.2; *p* = 1.15 × 10^-5^). An open possibility is that we find few non-MIKC proteins in our list of prospective tetramers only because these proteins have narrow expression patterns, thus making it more unlikely that four such proteins coincide in at least one tissue. Nevertheless, if non-MIKC proteins have an important role in the formation of functional tetramers, we expect that it will be easier to find sets of four co-expressed non-MIKC proteins if they take part in a tetramer-like subgraph than when they are connected in any other manner. We went back to the MADS_*exp*_ network and counted the number of four-node connection patterns comprising only non-MIKC proteins. We distinguished between tetramer-like subgraphs and all other subgraphs, on the one hand, and between co-expression in at least one tissue and no co-expression, on the other hand (Table 6). A Pearson’s *χ*^2^ test does not allow us to reject the null hypothesis that a tetramer-like connection pattern and joint expression of encoding genes are not associated (*χ*^2^ = 0.37, df = 1, *p* = 0.54). Thus, it is not easier to find sets of non-MIKC proteins with joint expression of their encoding genes when they are connected according to a tetramer-like subgraph.

**Table 6:** Tetramer-like subgraphs comprising only non-MIKC proteins are not more likely to be co-expressed than other subgraphs

**Subgraphs**	**Not co-expressed (*A*)**	**Co-expressed (*J*)**	***J*/*A***
Not tetramer-like	80	97	1.2125
Tetramer-like	5	4	0.8

Taken together, the results presented in this section do not support that non-MIKC proteins have an important role in the formation of functional tetramers. Rather, they support that MIKC proteins are predominantly, perhaps exclusively, involved in the formation of MADS domain protein tetramers.

### Retrodiction of experimental results

Experimental studies have found several examples of MADS domain proteins that assemble into functional tetramers (see Table S4 in Additional file 1). We checked whether those experimentally supported tetramers appear in our list of prospective tetramers (Additional file 2).

Based on genetic data Gregis et al. infer that the proteins AP1, AGAMOUS-LIKE 24 (AGL24), SHORT VEGETATIVE PHASE (SVP) and CAULIFLOWER (CAL) participate in a functional tetramer [44]. Indeed, this set of proteins appears in our list of prospective tetramers, thus supporting that this set of proteins may build a functional tetramer. The story is not as straightforward for the remaining experimentally-supported tetramers for the following reasons: First, several of these tetramers include the protein APETALA3 (AP3). In the data sets from which we built our network, AP3 only interacts with PISTILLATA (PI). Thus, in our study, AP3 can not take part in a tetramer-like subgraph, defined by each node having two interactions with different proteins. Second, one tetramer included a protein, AGAMOUS-LIKE 30 (AGL30), that has no interactions in our data set. Third, a protein appears twice in a validated tetramer. Thus, a three-node subgraph would represent one such tetramer. This occurs for AG, AP1 and SEP3. The latter is the protein that appears in most of the validated tetramers.

In order to partially overcome the limitations mentioned in the previous paragraph, we considered that SEP1 is a very close paralogue of SEP3 [4,5]. Moreover, SEP1 and SEP3 are highly redundant [42], and they share many of the proteins with which they interact [15,25]. Therefore, we assumed that SEP1 could replace one SEP3 instance in a tetramer. It is noteworthy that for all cases where the only limitation was that SEP3 appeared twice in a validated tetramer, we found a match in the list of prospective tetramers where SEP1 replaced one of the SEP3 instances.

Loss of function mutations of several different genes that encode for MADS domain proteins do not display obvious phenotypic alterations. The reason is that they are redundant with other MADS domain proteins so that phenotypic effects appear only in multiple mutant combinations [8]. Identification of redundant genes may not be easy. Thus, we attempted to retrodict experimentally validated combinations of redundant genes as a means to evaluate the potential usefulness of our results as an aid for the design of new experiments.

First we searched in our list of prospective tetramers for all those that included a protein of our interest, X. For one of the X-including prospective tetramers, e.g. X-A-B-C, we scanned again the whole list of prospective tetramers to identify all those protein sets that share all proteins with the exception of X. In other words, we identified sets Y-A-B-C, Z-A-B-C, and so on. We preserved each such set only if the gene encoding for the protein that replaced X was co-expressed, in at least one tissue, with those genes of all four proteins in the original X-including prospective tetramer. Gene expression was determined from microarray expression data [40] (see Methods). We repeated the above for each of the remaining X-including prospective tetramers. We expected that proteins that replaced X most often in the prospective tetramers were more likely to be redundant with X. Indeed, we found that this was the case, as we describe below.

Pelaz and collaborators found that the SEP1-3 proteins are highly redundant [42]. After following the algorithm described in the preceding paragraph, we found that SEP3 most often replaces SEP1 in prospective tetramers, both SEP1 and SEP3 substitute SEP2 more frequently, and SEP1 takes the place of SEP3 with greatest probability. Another relevant example concerns the protein CAL. Loss of function *cal* mutants do not display an obvious phenotype, unless combined with the *ap1* mutation [45]. In our analysis, there are three proteins that replace CAL most often in the same number of prospective tetramers: AP1, AGL6, and SEP1. Thus, also in this case we could identify the protein, AP1, that substitutes for the function of CAL. To our knowledge, there is no experimental evidence that loss of function *agl6* or *sep1* mutations could enhance the *cal* phenotype, but this remains as an open possibility and a prediction derived from our analyses. Finally, the SHATTERPROOF1/2 (SHP1/2) proteins are also redundant [46]. Again, SHP2 is the protein that substitutes SHP1 most times in our list of prospective tetramers. These results support that our list of prospective tetramers and simple algorithms as that described above may be of value to infer relationships among MADS domain proteins and design experiments accordingly.

### Homodimers in the formation of tetramers

So far, we have addressed how the patterns of interactions within sets of four different proteins may be indicative of MADS-domain protein heterotetramers. Hence, our analyses have left out of the picture those tetramers that incorporate two copies of the same protein. Our method, based on the analysis of pairwise interaction patterns, is unable to identify some of such tetramers. Consider for instance a hypothetical protein A that forms two kinds of dimers, one with protein B and other with protein C. The A molecule in the A-B dimer binds C in the A-C dimer, and the A molecule in the A-C dimer binds B in the other dimer to form a tetramer. In our network we would only observe interactions between A and B and between A and C (B-A-C), which is a connection pattern that appears in all non-disjoint 3 node sets. This argument is even stronger when we consider a tetramer in which two A-B dimers form a tetramer through interactions between A and B molecules in different dimers. Here, we would be unable to distinguish the interaction pattern associated to this tetramer (A-B) from any pairwise interaction in our network. Thus, our method is clearly not useful to associate a connection pattern to some of the possible tetramers that include two or more copies of a single protein. However, there are some cases where this association can be accomplished. These cases involve tetramers that incorporate homodimers.

As assumed for heterotetramers, we expect that each protein molecule in a tetramer that incorporates homodimers also binds two other protein molecules. The only difference to heterotetramers is that one such interaction must occur between the identical protein molecules that form the homodimer. Consider first a tetramer that contains a single homodimer. The corresponding 3-node subgraph is a triangle in which each node binds the other two nodes, and at least one node has a self interaction. For brevity, we will refer to 3-node connection patterns that embed this subgraph as three-node tetramer patterns (3NTP). Now consider a tetramer that incorporates two different homodimers. In this case, the subgraph corresponds to a pair of interacting nodes, each with self-interactions. We will call these subgraphs two-node tetramer patterns (2NTP). Subgraphs that include either of these interaction patterns are not significantly abundant in the MADS network when compared to randomized networks with the same connectivity distribution. However, it must be taken into account that few MADS domain proteins self-interact. Indeed, self-interactions occur significantly less often in the MADS network (6 self-interactions) than in randomized networks (a median of 10 self-interactions) (*p* = 0.045). It is thus relevant comparing the counts of these subgraphs in the MADS network to those in randomized networks with the same number of self-interactions as the MADS network. In this case, the number of occurrences of 2NTP’s and 3NTP’s is significantly higher in the MADS network. Out of 10^4^ randomized networks with the same connectivity distribution and the same number of nodes, interactions and self-interactions as the MADS network, there are only two that have as many 2NTP’s as the MADS network (*p* = 0.0002) and none with as many 3NTP’s as the MADS network (*p* < 10^-4^). While the MADS network has 14 and 235 2NTP’s and 3NTP’s, respectively, randomized networks have a median of 8 2NTP’s and 160 3NTP’s. Hence, when we control for the occurrence of self-interactions, subgraphs associated to homodimer-including tetramers are overrepresented in the MADS network.

We also evaluated whether the mRNA coding for proteins in homodimer-including tetramers is co-expressed more often than for proteins in other subgraphs. The MADS_*exp*_ network contains 14 2NTP’s, all of which have proteins with jointly expressed mRNA in at least one tissue. In contrast, there are 147 two-node subgraphs that are not 2NTP’s in the MADS network, but only for 131 of them the pair of proteins are jointly expressed, as inferred from RNA expression patterns. In other words, while proteins in 100% (14/14) 2NTP’s are jointly expressed, proteins in only 89% (131/147) of non-2NTP’s are co-expressed. The difference is not statistically significant according to a Pearson’s *χ*^2^ test (*χ*^2^ = 1.69; *df* = 1; *p* = 0.193). However, this lack of significance may not be conclusive because of the low number of 2NTP’s in the MADS network.

As for 3NTP’s, there are 229 in the MADS network, and 213 of them are composed of proteins with mRNA co-expressed in one or more tissues. At the same time, the MADS network has 922 non-3NTP 3-node subgraphs, but only 752 of them are jointly expressed. Hence, the proteins in 93% of 3NTP’s are co-expressed in at least one tissue, while proteins in only 81% (752/922) of non-3NTP’s are expressed jointly. Indeed, there is a significant statistical association between joint expression and 3NTP’s (*χ*^2^ = 17.75; *df* = 1; *p* = 2.5 × 10^-5^).

## Discussion

Large and complex networks of distinct kinds of molecules orchestrate developmental mechanisms. High throughput tools, such as microarray experiments [40,47] or large- scale yeast two-hybrid studies [15,48,49], are enabling the empirical identification of such networks. In order to study those systems, computational and mathematical tools are being used to organize and test the sufficiency of available experimental information to reproduce observed phenomena [50,51].

Many networks share global features, such as a power-law connectivity distribution, or the property of having short paths between any two elements [52-55]. But several types of networks are different at an intermediate scale [27,56]. For example, some networks contain certain subgraphs that are overrepresented with respect to randomly generated networks with the same number of nodes, edges and degree distribution [26-28]. Hence, a particular subgraph composition may characterize a certain kind of network [28]. Such deviations from randomness may arise as a side-effect of a network’s generative rules [57]. For example, Artzy-Randrup and collaborators considered a set of nodes, each fixed in a spatial location. Connecting such nodes in a random fashion, but with a bias towards nearer nodes, produces a statistically significant abundance of the same subgraphs that are overrepresented in the network of spatially-aggregated neurons of the nematod *Caenorhabditis elegans*[58]. Another example concerns gene regulatory networks. Kuo et al. analyzed a gene regulatory network model in which interactions are defined by complementarity of binary ‘protein’ and ‘cis-regulatory’ sequences. They found that neutral processes, like duplication and mutation, suffice to produce an abundance of subgraphs similar to that of regulatory networks in yeast and *Escherichia coli*[59]. However, in other cases, the abundance of some subgraphs may be attributed to adaptive optimization of the biological process controlled by the network, if the subgraph’s connection pattern confers an advantage to the organism [27,60,61]. This is very likely the case where alternative neutral scenarios, like duplication of entire subgraphs, have been discarded [61], or for subgraphs with dynamic properties that may increase the efficiency of tasks like information transfer [29,30]. In sum, because neutral and adaptive explanations may be valid under different circumstances, the possible role of natural selection as an explanation for network structure should be tested for each case.

The formation of transcription factor tetramers is not exclusive of MADS domain proteins. Several transcriptional regulators do so, both in prokaryotes and eukaryotes. Pertinent examples include the lactose repressor LacI [62], Stat5 proteins [63], the IclR protein TtgV [64], bHLH proteins as in the Max-Myc heterotetramer [65], or proteins in the p53 family of transcription factors [66,67]. In the present work we show that subgraphs compatible with MADS domain protein heterotetramers appear significantly more often than as expected by chance in the *Arabidopsis* network of physical interactions between these proteins. Formation of transcription factor oligomers may increase specificity of protein-DNA interaction [62] or allow synergistic binding to distinct DNA motifs [63]. For these reasons, tetramer formation in MADS domain proteins could be beneficial [19]. In fact, several of our observations suggest that the abundance of tetramer-like subgraphs is due to the advantages that the formation of tetramers of MADS domain proteins may confer to plants. First, the same kind of subgraphs is not significantly abundant in a network of physical interactions for a different family of plant transcription factors. These results are consistent with the hypothesis that the abundance of tetramer-like subgraphs can not be attributed to a common mechanism in the evolution of plant transcription factors. Moreover, we found that the mRNA of proteins in tetramer-like subgraphs is co-expressed more frequently than that of proteins in other subgraphs. Such an enhanced coordination of expression is expected if these subgraphs are associated to proteins that jointly exert their functions. One could think that sometimes gene duplication can increase the counts of what we call tetramer-like subgraphs, even without forming new tetramers. For instance, consider a protein X that can bind either protein Y or Z. If the gene coding for X is duplicated, then the new gene X’ would also have interactions with Y and Z. The resulting connection pattern would be a tetramer-like subgraph that does not correspond to a real functional tetramer. However, it must also be acknowledged that other equally probable duplications increase the frequencies of other subgraphs. Take as an example a set of four proteins in which A binds B, B binds C and C binds D. This interaction pattern is the linear subgraph A-B-C-D. Say D duplicates to produce D’ and D”. Then we would get twice the linear subgraph: A-B-C-D’ and A-B-C-D”. This argument is equally valid for duplication of the remaining three nodes. Although many subgraphs can increase their frequency because of duplication, not all of them are significantly abundant in the MADS network, when compared to what is expected by chance. Moreover, it is also noteworthy that joint-expression, as inferred from mRNA patterns, occurs significantly more often for tetramer-like subgraphs but not other subgraphs. In sum, our results suggest that formation of functional tetramers, as proposed by the ‘quartet model’ [17,18], is a property of a large group of *Arabidopsis* MADS domain proteins, and is not restricted to the few experimentally validated cases [20-24].

In addition, we found that proteins in the monophyletic MIKC clade that take part in a tetramer-like subgraph will very likely be co-expressed in at least one *Arabidopsis* tissue. In contrast, we found no association between tetramer-like subgraphs and joint expression for sets of MADS domain proteins outside of the MIKC clade. These observations further support that functional tetramers comprise MIKC proteins exclusively. Our results, albeit forcefully based on a small number of non-MIKC proteins with mRNA expression patterns, are statistically sound. The prediction that MIKC proteins are the ones mainly involved in the formation of functional tetramers is consistent with the fact that all MADS domain tetramers that have been experimentally verified up to now comprise only MIKC proteins [20-24]. It well may be that the presence of the K domain, a specific trait of MIKC proteins that evolved along the plant lineage [12], facilitates tetramer formation. In fact, many of the interactions between proteins in tetramers occur through their K domains, as described in the Background section [14,20,21,33,34].

We also used the AtGenExpress data [40] in combination with the protein interaction information to extract protein sets in tetramer-like subgraphs for which the encoding genes are jointly expressed in at least one *Arabidopsis* tissue. In that manner we built a list of prospective tetramers (Additional file 2). Despite the overlap in expression, we cannot exclude that some of such protein sets do not represent functional tetramers occurring *in planta*. One likely cause would be that some interactions in a prospective tetramer may be mutually exclusive. This would occur if one protein binds two other proteins in the set through the same protein domain. Furthermore, the list of prospective tetramers is still rather long, though it is not unreasonable to think that a large number of functional tetramers occur in plants. However, it should be acknowledged that this is a major reduction of the search space, from 79^4^ ∼ 39 × 10^6^, considering only the 79 proteins in the MADS network, to just over a thousand. Moreover, analyses of this list of prospective tetramers allow the retrodiction of several experimental results, thus demonstrating its predictive value.

Most of the interactions with which we assembled the MADS network are pairwise, as they were mostly derived from yeast two-hybrid studies. One could argue that pairwise interactions do not provide information regarding higher-order protein complexes. Indeed, this would be an issue if each of the interactions between proteins in a tetramer only occurred in the presence of three or more of the tetramer’s constituent proteins. For the sake of simplicity, we assumed this was not the case. Notwithstanding, there is substantial evidence that backs up our assumption. This evidence concerns yeast two-hybrid data that supports the majority of the interactions that we derived from yeast three-hybrid assays. Moreover, we found the same significantly abundant subgraphs in our MADS network and in a network that excludes interactions supported only by yeast three-hybrid data (Tables S2 and S3 in Additional file 1). These observations substantiate our claim that pairwise interactions have a prevalent role in the formation of higher-order complexes of MADS domain proteins.

## Conclusions

In sum, our work supports that the formation of functional tetramers is a widespread property of plant MADS domain transcription factors, specifically of MIKC proteins. Experimental research will be required to settle the issue, which is critical in the understanding of several mechanisms in plant development at the molecular level. However, the computational analysis that we put forward facilitates this endeavor by pointing those protein sets with higher chances of participating in functional tetrameric complexes.

## Methods

### Assemblage of the MADS network

In the MADS network, nodes represent the different MADS domain proteins in the plant *Arabidopsis thaliana* and links joining two nodes represent the potential of two proteins to interact physically. The main source of data to assemble the network is the study by de Folter et al. [15], that provides a semi-exhaustive map of protein-protein interactions between MADS domain proteins based on yeast two-hybrid assays. Another important source of information is a series of yeast three-hybrid assays for *Arabidopsis* MADS domain proteins [25]. We took additional experimental evidence from the sources listed in Table S1 in Additional file 1. We did not consider those *Arabidopsis* MADS domain proteins for which we found no evidence of protein-protein interactions.

For every positive yeast two-hybrid result, an interaction between the two proteins involved was added. For every positive yeast three-hybrid experiment, two new interactions were added. For example, consider the yeast three-hybrid experiment involving the hypothetical proteins A, B, and C, where A was fused to a DNA-binding domain and C to a transactivation domain. If the result was positive and there was no yeast two-hybrid experiment indicating an interaction between A and C, then the interactions between A and B, and between B and C were included.

In order to obtain the MADS_*exp*_ network, we eliminated from the MADS network all those proteins that lack expression data in the AtGenExpress data set. We also discarded those proteins that only interacted with proteins that lack expression data, as they would be devoid of interactions in the MADS_*exp*_ network. We followed an analogous procedure to obtain the MADS_*At*-*TAX*_ network.

### Connection patterns

We built a catalogue with all possible manners of connecting up to four nodes in a single component (Figure 2). In other words, the connection patterns that we follow are not disjoint. We accomplished this by exhaustive enumeration and dismissing those connection patterns that do not form a single component and those that were isomorphic to connection patterns already in the catalogue. Two subgraphs are isomorphic if there is a one-to-one correspondence between their node sets which preserves adjacency [68].

In order to count the number of *n*-node connection patterns in a given network, we determined for each possible *n*-node set in the network whether it formed a single component. If that was the case, we searched for its isomorphic subgraph in our catalogue to increase its count.

### Network randomization

In order to obtain an ensemble of randomized networks comparable to the MADS network we performed the ‘switching’ algorithm described by Milo et al. [27]. For each randomized network in the ensemble we started with the original MADS network as seed, and iteratively we randomly picked two connections and exchanged two of their ends. For example, assume we pick connections joining proteins A and B, on one hand, and proteins C and D, on the other. We then replaced those connections for one joining A and D, and another joining C and B. In this manner, each node preserves its number of connections, but now they are linked to different nodes. We switched pairs of connections *s* times, with *s* taken with an uniform probability distribution from the interval [100*E*, 200*E*]. Here, *E* stands for the number of connections in the MADS network. The large value of *s* guarantees that all traces of the original structure of the MADS network is lost in its randomized counterparts. At the same time, this procedure preserves the number of connections that each node has, and thus the network’s connectivity distribution.

For building randomized networks that, in addition to maintaining the connectivity distribution, preserve the number of self-interactions we modified slightly the procedure described above. The modification merely consisted on prohibiting both the formation and loss of self-interactions.

### Expression data

We obtained expression data for *Arabidopsis* MADS-box genes from the developmental data set of the AtGenExpress expression atlas (http://weigelworld.org/resources/microarray/AtGenExpress) [40]. We excluded from our analyses those samples that came from mutant backgrounds. We used gcRMA to obtain expression data. We considered a certain gene in a given sample as expressed only if the log_2_ of its average expression was greater than a pre-specified threshold. We followed Immink et al. [25] and set this threshold to four for all of our analyses.

To build the MADS_*At*-*TAX*_ network, we obtained mean-normalized values from the developmental set in the At-TAX database (http://jsp.weigelworld.org/tileviz/tileviz.jsp) [41].

## Competing interests

The authors declare that they have no competing interests.

## Authors’ contributions

CE-S performed all computational and statistical analyses. SdF, RGHI and GCA provided data. CE-S, SdF and ERA-B designed the study and drafted the manuscript. RGHI and GCA suggested additional analyses. All authors read and approved the final manuscript.

## Supplementary Material

Additional file 1 **Additional tables.** This file contains additional tables S1–S4. Table S1 provides references for each interaction in the MADS network. Tables S2 and S3 present results analogous to those of Tables 1 and 2, respectively, but for a network that considers only interactions from a large-scale yeast two-hybrid study [15]. Table S4 presents a list of experimentally validated tetramers of MADS domain proteins. Click here for file

Additional file 2 **List of prospective tetramers.** This additional text file contains a list of those proteins in four-node tetramer-like subgraphs of which the encoding genes are jointly expressed in at least one *Arabidopsis* tissue. Click here for file
