# Supplementary material for: Tetramer formation in Arabidopsis MADS domain proteins: analysis of a protein-protein interaction network
Source: BMC Syst Biol. 2014 Jan 27;8:9. doi: 10.1186/1752-0509-8-9 (PMC3913338; doi:10.1186/1752-0509-8-9)
Supplement: Additional file 1 — Additional tables. This file contains additional tables S1–S4. Table S1 provides references for each interaction in the MADS network. Tables S2 and S3 present results analogous to those of Tables 1 and 2, respectively, but for a network that considers only interactions from a large-scale yeast two-hybrid study [15]. Table S4 presents a list of experimentally validated tetramers of MADS domain proteins. [file 1752-0509-8-9-S1.pdf]

Table S1: Interactions between MADS domain proteins in *A. thaliana*

Interaction		References
SEP1	*AG*	de Folter et al. (2005); Fan et al. (1997)
SEP1	*SHP1*	de Folter et al. (2005); Immink et al. (2009)^T
SEP2	*AG*	de Folter et al. (2005); Fan et al. (1997)
SHP2	*SEP1*	de Folter et al. (2005); Immink et al. (2009)^T
AGL6	*AG*	Immink et al. (2009)^T; Fan et al. (1997)
AGL6	*SEP1*	de Folter et al. (2005)
AGL6	*SHP2*	de Folter et al. (2005)
AP1	*SEP1*	de Folter et al. (2005); Immink et al. (2009)^T
AP1	*AGL6*	de Folter et al. (2005)
FUL	*AG*	de Folter et al. (2005)
FUL	*SEP1*	de Folter et al. (2005)
FUL	*AGL6*	de Folter et al. (2005)
SEP3	*AG*	de Folter et al. (2005); Immink et al. (2009)^T; Favaro et al. (2003)^{D,T}; Fan et al. (1997)
SEP3	*SHP1*	de Folter et al. (2005); Immink et al. (2009)^T; Favaro et al. (2003)^{D,T}
SEP3	*SEP1*	Immink et al. (2009)^T
SEP3	*SHP2*	de Folter et al. (2005); Immink et al. (2009)^T; Favaro et al. (2003)^{D,T}
SEP3	*AGL6*	de Folter et al. (2005)
SEP3	*AP1*	de Folter et al. (2005); Immink et al. (2009)^T; Pelaz et al. (2001)
SEP3	*FUL*	de Folter et al. (2005); Immink et al. (2009)^T
SEP3	*SEP3*	Melzer et al. (2009)
CAL	*SEP3*	Pelaz et al. (2001)
STK	*SEP1*	de Folter et al. (2005)
STK	*SEP3*	de Folter et al. (2005); Immink et al. (2009)^T; Favaro et al. (2003)^{D,T}
AGL12	*SEP1*	de Folter et al. (2005)
AGL13	*AG*	de Folter et al. (2005)
AGL13	*SHP1*	de Folter et al. (2005)
AGL14	*AG*	Immink et al. (2009)^T
AGL14	*SHP1*	Immink et al. (2009)^T
AGL14	*SHP2*	Immink et al. (2009)^T
AGL14	*FUL*	de Folter et al. (2005)
AGL14	*SEP3*	Immink et al. (2009)^T
AGL15	*AG*	de Folter et al. (2005)
AGL15	*SHP1*	de Folter et al. (2005)
AGL15	*SHP2*	de Folter et al. (2005)
AGL15	*AGL6*	de Folter et al. (2005)
AGL15	*AP1*	de Folter et al. (2005); Immink et al. (2009)^T

Table S1 – continued from previous page

Interaction		References
AGL15	*STK*	de Folter et al. (2005); Immink et al. (2009)^T
AGL15	*AGL15*	de Folter et al. (2005)
AGL16	*AG*	de Folter et al. (2005)
AGL16	*SHP1*	de Folter et al. (2005)
AGL16	*SEP1*	de Folter et al. (2005)
AGL16	*SEP2*	de Folter et al. (2005)
AGL16	*SHP2*	de Folter et al. (2005)
AGL16	*AGL6*	de Folter et al. (2005)
AGL16	*AP1*	de Folter et al. (2005)
AGL16	*SEP3*	de Folter et al. (2005); Immink et al. (2009)^T
AGL16	*STK*	de Folter et al. (2005)
AGL16	*AGL12*	de Folter et al. (2005)
AGL16	*AGL14*	de Folter et al. (2005)
AGL16	*AGL15*	de Folter et al. (2005)
AGL16	*AGL16*	de Folter et al. (2005)
AGL17	*AGL16*	de Folter et al. (2005)
AGL19	*AGL17*	Immink et al. (2009)^T
SOC1	*SHP1*	de Folter et al. (2005)
SOC1	*SEP1*	de Folter et al. (2005); Immink et al. (2009)^T
SOC1	*SEP4*	de Folter et al. (2005)
SOC1	*SEP2*	de Folter et al. (2005); Immink et al. (2009)^T
SOC1	*SHP2*	de Folter et al. (2005)
SOC1	*AGL6*	de Folter et al. (2005); Immink et al. (2009)^T
SOC1	*AP1*	de Folter et al. (2005); Immink et al. (2009)^T; Pelaz et al. (2001)
SOC1	*FUL*	de Folter et al. (2005); Immink et al. (2009)^T
SOC1	*SEP3*	de Folter et al. (2005); Immink et al. (2009)^T
SOC1	*CAL*	de Folter et al. (2005); Pelaz et al. (2001)
SOC1	*AGL12*	de Folter et al. (2005)
SOC1	*AGL13*	de Folter et al. (2005)
SOC1	*AGL14*	de Folter et al. (2005); Immink et al. (2009)^T
SOC1	*AGL15*	de Folter et al. (2005)
SOC1	*AGL16*	de Folter et al. (2005)
SOC1	*AGL17*	de Folter et al. (2005); Immink et al. (2009)^T
SOC1	*AGL19*	de Folter et al. (2005); Immink et al. (2009)^T
SOC1	*SOC1*	de Folter et al. (2005)
AGL21	*AG*	de Folter et al. (2005)
AGL21	*SHP1*	de Folter et al. (2005)
AGL21	*SEP1*	de Folter et al. (2005)
AGL21	*SEP2*	de Folter et al. (2005)
AGL21	*SHP2*	de Folter et al. (2005)

Table S1 – continued from previous page

Interaction		References
AGL21	*AGL6*	de Folter et al. (2005)
AGL21	*AP1*	de Folter et al. (2005)
AGL21	*FUL*	de Folter et al. (2005)
AGL21	*SEP3*	Immink et al. (2009)^T
AGL21	*STK*	de Folter et al. (2005)
AGL21	*AGL12*	de Folter et al. (2005)
AGL21	*AGL13*	de Folter et al. (2005)
AGL21	*AGL15*	de Folter et al. (2005)
AGL21	*AGL16*	de Folter et al. (2005)
AGL21	*AGL17*	de Folter et al. (2005)
AGL21	*AGL19*	de Folter et al. (2005)
AGL21	*SOC1*	de Folter et al. (2005); Immink et al. (2009)^T
AGL21	*AGL21*	de Folter et al. (2005)
SVP	*SEP1*	de Folter et al. (2005)
SVP	*AGL6*	de Folter et al. (2005)
SVP	*AP1*	de Folter et al. (2005); Pelaz et al. (2001)
SVP	*SEP3*	de Folter et al. (2005); Immink et al. (2009)^T
SVP	*CAL*	Pelaz et al. (2001)
SVP	*AGL15*	de Folter et al. (2005)
SVP	*SOC1*	de Folter et al. (2005)
SVP	*AGL21*	de Folter et al. (2005)
AGL24	*AG*	de Folter et al. (2005)
AGL24	*SHP1*	de Folter et al. (2005)
AGL24	*SEP1*	de Folter et al. (2005); Immink et al. (2009)^T
AGL24	*AGL6*	de Folter et al. (2005)
AGL24	*AP1*	de Folter et al. (2005); Immink et al. (2009)^T; Pelaz et al. (2001)
AGL24	*FUL*	de Folter et al. (2005); Immink et al. (2009)^T
AGL24	*SEP3*	de Folter et al. (2005); Immink et al. (2009)^T
AGL24	*CAL*	Pelaz et al. (2001)
AGL24	*AGL14*	de Folter et al. (2005)
AGL24	*AGL15*	de Folter et al. (2005)
AGL24	*AGL16*	de Folter et al. (2005)
AGL24	*SOC1*	de Folter et al. (2005); Immink et al. (2009)^T
AGL24	*AGL21*	de Folter et al. (2005)
AGL24	*AGL24*	de Folter et al. (2005)
FLM	*AP1*	Pelaz et al. (2001)
ABS	*SEP1*	de Folter et al. (2005); Immink et al. (2009)^T
ABS	*SEP2*	de Folter et al. (2005); Immink et al. (2009)^T
ABS	*SEP3*	de Folter et al. (2005); Immink et al. (2009)^T
AGL36	*AGL28*	de Folter et al. (2005)

Table S1 – continued from previous page

Interaction		References
PHE1	*AGL28*	de Folter et al. (2005)
PHE2	*AGL28*	de Folter et al. (2005)
AGL39	*AG*	de Folter et al. (2005)
AGL39	*AGL6*	de Folter et al. (2005)
AGL39	*AP1*	de Folter et al. (2005)
AGL39	*FLM*	de Folter et al. (2005)
AGL39	*PHE1*	de Folter et al. (2005)
AGL40	*PHE1*	de Folter et al. (2005)
AGL40	*PHE2*	de Folter et al. (2005)
AGL42	*SEP1*	de Folter et al. (2005)
AGL42	*AGL6*	de Folter et al. (2005)
AGL42	*AGL16*	de Folter et al. (2005)
AGL42	*SOC1*	de Folter et al. (2005); Immink et al. (2009)^T
AGL42	*AGL21*	de Folter et al. (2005)
ANR1	*SEP3*	Immink et al. (2009)^T
ANR1	*AGL16*	de Folter et al. (2005)
ANR1	*SOC1*	de Folter et al. (2005)
ANR1	*AGL21*	de Folter et al. (2005)
AGL45	*AGL40*	de Folter et al. (2005)
AGL49	*AGL39*	de Folter et al. (2005)
AGL52	*AGL39*	de Folter et al. (2005)
AGL53	*AGL6*	de Folter et al. (2005)
AGL53	*AGL16*	de Folter et al. (2005)
AGL55	*AGL52*	de Folter et al. (2005)
AGL56	*AGL52*	de Folter et al. (2005)
AGL62	*AGL36*	de Folter et al. (2005)
AGL62	*PHE1*	de Folter et al. (2005)
AGL62	*PHE2*	de Folter et al. (2005)
AGL63	*AGL16*	de Folter et al. (2005)
AGL64	*AGL48*	de Folter et al. (2005)
AGL66	*AGL6*	de Folter et al. (2005)
AGL66	*AGL65*	de Folter et al. (2005); Immink et al. (2009)^T
AGL71	*SEP1*	de Folter et al. (2005)
AGL71	*SOC1*	de Folter et al. (2005)
AGL73	*AGL26*	de Folter et al. (2005); Immink et al. (2009)^T
AGL73	*AGL43*	de Folter et al. (2005)
AGL73	*AGL52*	de Folter et al. (2005)
AGL73	*AGL53*	de Folter et al. (2005)
AGL77	*AGL39*	de Folter et al. (2005)
AGL77	*AGL64*	de Folter et al. (2005)

Table S1 – continued from previous page

Interaction		References
AGL77	*AGL73*	de Folter et al. (2005)
AGL78	*AGL6*	de Folter et al. (2005)
AGL78	*AGL39*	de Folter et al. (2005)
AGL78	*AGL55*	de Folter et al. (2005); Immink et al. (2009)^T
AGL78	*AGL56*	de Folter et al. (2005)
AGL78	*AGL73*	de Folter et al. (2005)
AGL78	*AGL77*	de Folter et al. (2005)
AGL80	*AGL26*	Immink et al. (2009)^T
AGL80	*AGL62*	de Folter et al. (2005)
AGL82	*AGL39*	de Folter et al. (2005)
AGL82	*AGL66*	de Folter et al. (2005)
AGL83	*AGL53*	de Folter et al. (2005)
AGL83	*AGL54*	de Folter et al. (2005)
AGL84	*AGL26*	de Folter et al. (2005)
AGL84	*AGL43*	de Folter et al. (2005)
AGL84	*AGL52*	de Folter et al. (2005); Immink et al. (2009)^T
AGL84	*AGL53*	de Folter et al. (2005)
AGL84	*AGL76*	de Folter et al. (2005)
AGL84	*AGL77*	de Folter et al. (2005)
AGL84	*AGL78*	de Folter et al. (2005)
AGL84	*AGL79*	Immink et al. (2009)^T
AGL86	*AGL6*	de Folter et al. (2005); Immink et al. (2009)^T
AGL86	*AP1*	de Folter et al. (2005)
AGL86	*AGL23*	de Folter et al. (2005)
AGL86	*AGL28*	de Folter et al. (2005)
AGL86	*AGL40*	de Folter et al. (2005)
AGL86	*AGL62*	de Folter et al. (2005)
AGL86	*AGL65*	de Folter et al. (2005)
AGL86	*AGL82*	de Folter et al. (2005)
AGL89	*AGL73*	de Folter et al. (2005)
AGL89	*AGL84*	de Folter et al. (2005)
AGL90	*AGL40*	de Folter et al. (2005)
AGL90	*AGL62*	de Folter et al. (2005)
AGL90	*AGL66*	de Folter et al. (2005)
AGL90	*AGL86*	de Folter et al. (2005)
AGL92	*AGL6*	de Folter et al. (2005)
AGL92	*AP1*	de Folter et al. (2005)
AGL92	*AGL23*	de Folter et al. (2005)
AGL92	*AGL40*	Immink et al. (2009)^T
AGL92	*AGL62*	de Folter et al. (2005); Immink et al. (2009)^T

Table S1 – continued from previous page

Interaction		References
AGL92	*AGL78*	de Folter et al. (2005); Immink et al. (2009)^T
AGL92	*AGL90*	de Folter et al. (2005)
AGL96	*AGL66*	de Folter et al. (2005)
AGL97	*AG*	de Folter et al. (2005)
AGL97	*AGL6*	de Folter et al. (2005)
AGL97	*AP1*	de Folter et al. (2005)
AGL97	*AGL26*	de Folter et al. (2005)
AGL97	*FLM*	de Folter et al. (2005)
AGL97	*PHE1*	de Folter et al. (2005)
AGL97	*PHE2*	Immink et al. (2009)^D
AGL97	*AGL43*	de Folter et al. (2005)
AGL97	*AGL52*	de Folter et al. (2005)
AGL97	*AGL53*	de Folter et al. (2005)
AGL97	*AGL62*	de Folter et al. (2005)
AGL97	*AGL65*	de Folter et al. (2005)
AGL97	*AGL76*	de Folter et al. (2005)
AGL97	*AGL77*	de Folter et al. (2005)
AGL97	*AGL78*	de Folter et al. (2005)
AGL97	*AGL79*	Immink et al. (2009)^D
AGL97	*AGL82*	de Folter et al. (2005)
AGL97	*AGL87*	de Folter et al. (2005)
AGL97	*AGL89*	de Folter et al. (2005)
AGL97	*AGL90*	de Folter et al. (2005)
AGL98	*SOC1*	Immink et al. (2009)^D
AGL99	*PHE1*	de Folter et al. (2005)
AGL99	*PHE2*	Immink et al. (2009)^D
AGL99	*AGL43*	de Folter et al. (2005)
AGL99	*AGL52*	de Folter et al. (2005)
AGL99	*AGL76*	de Folter et al. (2005)
AGL99	*AGL77*	de Folter et al. (2005)
AGL99	*AGL78*	de Folter et al. (2005); Immink et al. (2009)^T
AGL99	*AGL79*	Immink et al. (2009)^D
AGL99	*AGL82*	de Folter et al. (2005)
AGL101	*AGL55*	Immink et al. (2009)^T
AGL101	*AGL56*	Immink et al. (2009)^T
AGL101	*AGL73*	de Folter et al. (2005)
AGL101	*AGL84*	de Folter et al. (2005); Immink et al. (2009)^T
AGL101	*AGL97*	de Folter et al. (2005)
AGL101	*AGL99*	Immink et al. (2009)^T
AGL102	*AGL78*	de Folter et al. (2005)

Table S1 – continued from previous page

Interaction		References
AGL102	*AGL82*	de Folter et al. (2005)
AGL102	*AGL97*	Immink et al. (2009)^D
AGL102	*AGL99*	Immink et al. (2009)^D
AGL102	*AGL101*	Immink et al. (2009)^T
AGL103	*AGL39*	de Folter et al. (2005); Immink et al. (2009)^T
AGL103	*AGL40*	Immink et al. (2009)^T
AGL103	*AGL55*	de Folter et al. (2005); Immink et al. (2009)^T
AGL103	*AGL56*	de Folter et al. (2005); Immink et al. (2009)^T
AGL103	*AGL92*	de Folter et al. (2005); Immink et al. (2009)^T
AGL103	*AGL97*	de Folter et al. (2005)
AGL103	*AGL99*	de Folter et al. (2005); Immink et al. (2009)^T
AGL103	*AGL101*	Immink et al. (2009)^T
AGL103	*AGL102*	de Folter et al. (2005); Immink et al. (2009)^T
AGL104	*AGL65*	de Folter et al. (2005); Immink et al. (2009)^T
AGL104	*AGL97*	de Folter et al. (2005)
PI	*AP1*	Honma and Goto (2001)^T
PI	*SEP3*	Immink et al. (2009)^T; Honma and Goto (2001)^T
PI	*AP3*	Immink et al. (2009)^T; Honma and Goto (2001)^T
ABS-2	*SEP1*	de Folter et al. (2005); Immink et al. (2009)^T
ABS-2	*SEP2*	Immink et al. (2009)^T
ABS-2	*SEP3*	de Folter et al. (2005); Immink et al. (2009)^T
ABS-2	*AGL14*	Immink et al. (2009)^T
ABS-2	*AGL63*	Immink et al. (2009)^T
ABS-2	*AGL78*	Immink et al. (2009)^T
ABS-2	*AGL92*	de Folter et al. (2005); Immink et al. (2009)^T
ABS-2	*AGL97*	de Folter et al. (2005)
SEP4-2	*AP1*	de Folter et al. (2005); Immink et al. (2009)^T
SEP4-2	*FUL*	de Folter et al. (2005); Immink et al. (2009)^T
SEP4-2	*SOC1*	de Folter et al. (2005); Immink et al. (2009)^T
SEP4-2	*SVP*	Immink et al. (2009)^T
SEP4-2	*AGL66*	de Folter et al. (2005)
SEP4-2	*AGL86*	de Folter et al. (2005)
SEP4-2	*AGL92*	de Folter et al. (2005)
SEP4-2	*AGL97*	de Folter et al. (2005)
AGL74-2	*AGL49*	de Folter et al. (2005)
AGL74-2	*AGL52*	de Folter et al. (2005)
AGL74-2	*AGL78*	de Folter et al. (2005); Immink et al. (2009)^T
AGL74-2	*AGL79*	Immink et al. (2009)^T
AGL74-2	*AGL84*	Immink et al. (2009)^T
AGL74-2	*AGL101*	Immink et al. (2009)^T

Table S1 – continued from previous page

Interaction		References
AGL74-2	*AGL103*	de Folter et al. (2005); Immink et al. (2009)[T]
AGL74-N	*AG*	de Folter et al. (2005)
AGL74-N	*SHP1*	de Folter et al. (2005)
AGL74-N	*SEP4*	de Folter et al. (2005)
AGL74-N	*SHP2*	de Folter et al. (2005)
AGL74-N	*AGL6*	de Folter et al. (2005)
AGL74-N	*AP1*	de Folter et al. (2005)
AGL74-N	*CAL*	de Folter et al. (2005)
AGL74-N	*STK*	de Folter et al. (2005)
AGL74-N	*AGL12*	de Folter et al. (2005)
AGL74-N	*AGL13*	de Folter et al. (2005)
AGL74-N	*AGL17*	de Folter et al. (2005)
AGL74-N	*AGL26*	de Folter et al. (2005)
AGL74-N	*FLM*	de Folter et al. (2005)
AGL74-N	*ABS*	de Folter et al. (2005); Immink et al. (2009)[T]
AGL74-N	*AGL42*	de Folter et al. (2005)
AGL74-N	*AGL43*	de Folter et al. (2005)
AGL74-N	*AGL49*	de Folter et al. (2005)
AGL74-N	*AGL52*	de Folter et al. (2005)
AGL74-N	*AGL55*	de Folter et al. (2005)
AGL74-N	*AGL56*	de Folter et al. (2005)
AGL74-N	*AGL65*	de Folter et al. (2005); Immink et al. (2009)[T]
AGL74-N	*AGL71*	de Folter et al. (2005)
AGL74-N	*AGL72*	de Folter et al. (2005)
AGL74-N	*AGL77*	de Folter et al. (2005)
AGL74-N	*AGL78*	de Folter et al. (2005)
AGL74-N	*AGL79*	Immink et al. (2009)[D]
AGL74-N	*AGL82*	de Folter et al. (2005)
AGL74-N	*AGL87*	de Folter et al. (2005)
AGL74-N	*AGL90*	de Folter et al. (2005)
AGL74-N	*AGL96*	de Folter et al. (2005)
AGL74-N	*AGL101*	de Folter et al. (2005)
AGL74-N	*AGL102*	Immink et al. (2009)[D]
AGL74-N	*AGL103*	de Folter et al. (2005)
AGL74-N	*ABS-2*	de Folter et al. (2005)
AGL74-N	*SEP4-2*	de Folter et al. (2005)

Table S2: Abundance of frequent subgraphs in a network of MADS domain proteins. This network includes exclusively interactions reported in a large-scale yeast two hybrid study (de Folter et al., 2005). Subgraph counts are compared to those of 10^4 randomized networks with the same degree distribution.

Subgraph	N_{MADS}	Randomized networks				$p-$value
		Mean	Median	Std. dev.	Std. error	
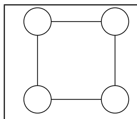	778	77.64	67	50.93	0.51	$< 10^{-4}$
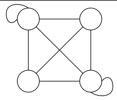	79	22.95	22	9.23	0.09	$< 10^{-4}$
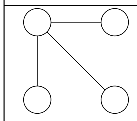	8331	1733.73	1491	1080.05	10.8	0.0001
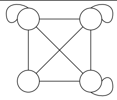	52	12.95	12	8.9	0.09	0.0002
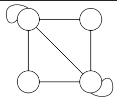	253	108.31	108	41.73	0.42	0.0009

Table S3: Abundance of connection patterns in a network of MADS domain proteins. This network includes exclusively interactions reported in a large-scale yeast two hybrid study (de Folter et al., 2005). Table shows results for connection patterns that forcefully include some interactions (solid line) but may or not contain other interactions (dashed lines).

Embedded subgraph	N_{MADS}	Randomized networks				$p-$value
		Mean	Median	Std. dev.	Std. error	
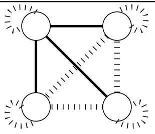	15032	14328.8	14278	506.41	5.06	0.095
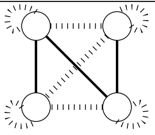	14983	17697.6	17693	482.23	4.82	1
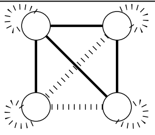	4348	5470.25	5470	365.436	3.65	0.999
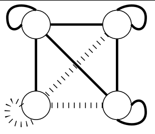	219	374.656	319	274.48	2.74	0.659
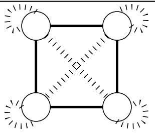	2126	1281.48	1280	92.39	0.92	$< 10^{-4}$

Table S4: Experimentally validated functional tetramers

Tetramer	Evidence	Status	Reference
AP3-PI-SEP3-AP1	Genetics and biochemistry	Confirmed	Honma and Goto (2001); Pelaz et al. (2001); Smaczniak et al. (2012)
AP3-PI-SEP3-AG	Genetics and biochemistry	Confirmed	Honma and Goto (2001); Smaczniak et al. (2012)
SEP3-AG-SEP3-AG	Biochemistry	Confirmed	Smaczniak et al. (2012)
SEP3-SEP3-AP3-PI	Biochemistry	Confirmed	Smaczniak et al. (2012)
SEP3-AP1-SEP3-AP1	Biochemistry	Confirmed	Smaczniak et al. (2012)
AG-SEP3-STK-SEP3	Genetics and biochemistry	Highly likely	Favaro et al. (2003)
AG-SEP3-SHP1-SEP3	Genetics and biochemistry	Highly likely	Favaro et al. (2003)
AG-SEP3-SHP2-SEP3	Genetics and biochemistry	Highly likely	Favaro et al. (2003)
STK-SEP3-STK-SEP3	Genetics and biochemistry	Highly likely	Favaro et al. (2003)
SHP1-SEP3-SHP2-SEP3	Genetics and biochemistry	Highly likely	Favaro et al. (2003)
STK-SEP3-ABS-SEP3	Genetics and biochemistry	Highly likely	de Folter et al. (2006); Mizzotti et al. (2012)
AG-SEP3-ABS-SEP3	Genetics and biochemistry	Highly likely	de Folter et al. (2006); Mizzotti et al. (2012)
ABS-SEP3-SHP1-SEP3	Genetics and biochemistry	Highly likely	de Folter et al. (2006); Mizzotti et al. (2012)
ABS-SEP3-SHP2-SEP3	Genetics and biochemistry	Highly likely	de Folter et al. (2006); Mizzotti et al. (2012)
AGL30-AGL65-AGL66-AGL104 †	Genetics and biochemistry	Highly likely	Verelst et al. (2007a,b)
AP1-AGL24-SVP-CAL	Genetics and biochemistry	Highly likely	(Gregis et al., 2008)

† This tetramer could not be searched in the list of prospective tetramers because in the data set that we used AGL30 has no interactions. However, it is noteworthy that the interactions that Verelst et al. (2007a,b) report are fully compatible with a tetramer-like subgraph.

References

de Folter, S., Immink, R., Kieffer, M., Parenicová, L., Henz, S., Weigel, D., Busscher, M., Kooiker, M., Colombo, L., Kater, M., Davies, B., and Angenent, G. (2005). Comprehensive interaction map of the Arabidopsis MADS box transcription factors. *Plant Cell*, 17:1424–1433.

de Folter, S., Shchennikova, A. V., Franken, J., Busscher, M., Baskar, R., Grossniklaus, U., Angenent, G. C., and Immink, R. G. H. (2006). A Bsister MADS-box gene involved in ovule and seed development in petunia and Arabidopsis. *Plant J.*, 47(6):934–946.

Fan, H. Y., Hu, Y., Tudor, M., and Ma, H. (1997). Specific interactions between the K domains of AG and AGLs, members of the MADS domain family of DNA binding proteins. *Plant J.*, 12(5):999–1010.

Favaro, R., Pinyopich, A., Battaglia, R., Kooiker, M., Borghi, L., Ditta, G., Yanofsky, M. F., Kater, M. M., and Colombo, L. (2003). MADS-box protein complexes control carpel and ovule development in Arabidopsis. *Plant Cell*, 15(11):2603–2611.

Gregis, V., Sessa, A., Colombo, L., and Kater, M. M. (2008). AGAMOUS-LIKE24 and SHORT VEGETATIVE PHASE determine floral meristem identity in Arabidopsis. *Plant J.*, 56(6):891–902.

Honma, T. and Goto, K. (2001). Complexes of MADS-box proteins are sufficient to convert leaves into floral organs. *Nature*, 409:525–529.

Immink, R. G., Tonaco, I. A., de Folter, S., Shchennikova, A., van Dijk, A. D., Busscher-Lange, J., Borst, J. W., and Angenent, G. C. (2009). SEPALLATA3: The 'glue' for MADS box transcription factor complex formation. *Genome Biol.*, 10:R24.

Melzer, R., Verelst, W., and Theißen, G. (2009). The class E floral homeotic protein SEPALLATA3 is sufficient to loop DNA in 'floral quartet'-like complexes in vitro. *Nucleic Acids Res.*, 37(1):144–157.

Mizzotti, C., Mendes, M. A., Caporali, E., Schnittger, A., Kater, M. M., Battaglia, R., and Colombo, L. (2012). The MADS box genes SEEDSTICK and ARABIDOPSIS Bsister play a maternal role in fertilization and seed development. *Plant J.*, 70(3):409–420.

Pelaz, S., Tapia-López, R., Alvarez-Buylla, E. R., and Yanofsky, M. F. (2001). Conversion of leaves into petals in Arabidopsis. *Curr. Biol.*, 11:182–184.

Smaczniak, C., Immink, R. G. H., Muiño, J. M., Blanvillain, R., Busscher, M., Busscher-Lange, J., Dinh, Q. D. P., Liu, S., Westphal, A. H., Boeren, S., Parcy, F., Xu, L., Carles, C. C., Angenent, G. C., and Kaufmann, K. (2012). Characterization of MADS-domain transcription factor complexes in Arabidopsis flower development. *Proc. Natl. Acad. Sci. USA*, 109(5):1560–1565.

Verelst, W., Saedler, H., and Münster, T. (2007a). MIKC* MADS-protein complexes bind motifs enriched in the proximal region of late pollen-specific Arabidopsis promoters. *Plant Physiol.*, 143(1):447–460.

Verelst, W., Twell, D., de Folter, S., Immink, R., Saedler, H., and Münster, T. (2007b). MADS-complexes regulate transcriptome dynamics during pollen maturation. *Genome Biol.*, 8(11):R249.
